# Comparability of PD‐L1 immunohistochemistry assays for non‐small‐cell lung cancer: a systematic review

**DOI:** 10.1111/his.14040

**Published:** 2020-03-24

**Authors:** Bregje M Koomen, Sushil K Badrising, Michel M van den Heuvel, Stefan M Willems

**Affiliations:** ^1^ Department of Pathology University Medical Center Utrecht Utrecht the Netherlands; ^2^ Department of Pulmonary Diseases Radboudumc Nijmegen the Netherlands

**Keywords:** immunohistochemistry, immunotherapy, non‐small‐cell lung cancer, predictive biomarker, programmed cell death‐ligand 1, systematic review

## Abstract

Programmed cell death ligand 1 (PD‐L1) immunohistochemistry is used to determine which patients with advanced non‐small‐cell lung cancer (NSCLC) respond best to treatment with PD‐L1 inhibitors. For each inhibitor, a unique immunohistochemical assay was developed. This systematic review gives an up‐to‐date insight into the comparability of standardised immunohistochemical assays and laboratory‐developed tests (LDTs), focusing specifically on tumour cell (TC) staining and scoring. A systematic search was performed identifying publications that assessed interassay, interobserver and/or interlaboratory concordance of PD‐L1 assays and LDTs in tissue of NSCLC patients. Of 4294 publications identified through the systematic search, 27 fulfilled the inclusion criteria and were of sufficient methodological quality. Studies assessing interassay concordance found high agreement between assays 22C3, 28‐8 and SP263 and properly validated LDTs, and lower concordance for comparisons involving SP142. A decrease in concordance, however, is seen with use of cut‐offs, which hampers interchangeability of PD‐L1 immunohistochemistry assays and LDTs. Studies assessing interobserver concordance found high agreement for all assays and LDTs, but lower agreement with use of a 1% cut‐off. This may be problematic in clinical practice, as discordance between pathologists at this cut‐off may result in some patients being denied valuable treatment options. Finally, five studies assessed interlaboratory concordance and found moderate to high agreement levels for various assays and LDTs. However, to assess the actual existence of interlaboratory variation in PD‐L1 testing and PD‐L1 positivity in clinical practice, studies using real‐world clinical pathology data are needed.

## Introduction

Since the approval of the first immune check‐point inhibitor in 2011,^1^, ^2^, ^3^ immunotherapy has become an important part of treatment for several forms of cancer. In patients with advanced non‐small‐cell lung cancer (NSCLC), treatment with programmed cell death‐1 (PD‐1) or programmed cell death‐ligand 1 (PD‐L1) inhibitors has become part of standard care. These patients may be treated with nivolumab or pembrolizumab, both anti‐PD‐1 check‐point inhibitors, or with an anti‐PD‐L1 check‐point inhibitor, i.e. atezolizumab or durvalumab.^4^, ^5^, ^6^, ^7^, ^8^, ^9^ Some of these drugs may only be prescribed to patients who show PD‐L1 expression in at least 1% or 50% of tumour cells, measured with immunohistochemistry (IHC).^10^, ^11^, ^12^ Immunohistochemical PD‐L1 testing thus aids clinicians in treatment decision‐making.

For each immune check‐point inhibitor, however, a separate immunohistochemistry (IHC) PD‐L1 assay has been developed. The PD‐L1 IHC 22C3 PharmDx assay was used in clinical trials assessing efficacy of pembrolizumab, and is therefore Food and Drug Administration (FDA)‐approved and Conformité Européenne (CE)‐marked as a companion diagnostic for prescription of this drug.^8^, ^13^, ^14^ In a similar fashion, the PD‐L1 IHC 28‐8 PharmDx assay was FDA‐approved and CE‐marked as a complementary diagnostic for nivolumab,^15^, ^16^ while the PD‐L1 IHC SP142 assay became a complementary diagnostic for atezolizumab.^17^, ^18^ Finally, the PD‐L1 IHC SP263 assay was developed for durvalumab, but it has also received CE marking for identification of patients eligible for treatment with pembrolizumab and of patients most likely to benefit from treatment with nivolumab.^19^, ^20^

Using all these different assays to test for PD‐L1 expression in one pathology laboratory is not feasible. Not only would it be expensive and time‐consuming to run so many different tests for each patient, most laboratories will not have both staining platforms (i.e. Dako and Ventana/Roche) needed for these tests at their disposal. Furthermore, the number of tests that can be performed is restricted due to limited tissue availability.^21^ It is thus important to assess whether results from different assays are interchangeable. In addition, it should be assessed if laboratory‐developed tests (LDTs) can be used instead of the standardised PD‐L1 assays. In recent years, a multitude of studies examining these issues has been published, such as the Blueprint PD‐L1 IHC Assay Comparison Project^22^ or the harmonisation studies by Ratcliffe *et al*.,^23^ Rimm *et al*.^24^ and Scheel *et al*.^25^ Others, such as Büttner *et al*.,^19^ have reviewed the analytical performance of PD‐L1 IHC assays previously. Considering the abundance of studies that have been published on the subject, however, there is need for a systematic, comprehensive and up‐to‐date overview of the literature, which not only focuses on interassay and interobserver concordance, but also includes a review of interlaboratory concordance. Hence, the aim of this study was to systematically review all studies that assessed interassay, interobserver and/or interlaboratory concordance of PD‐L1 IHC assays and LDTs, and in so doing provide an updated insight into the comparability of these standardised assays and LDTs.

## Materials and methods

### Search Strategy

A systematic search of PubMed, Embase and Cochrane Library was performed, using the search terms ‘lung cancer’ and ‘PD‐L1’ with all relevant synonyms (see Table S1). Only these two terms were used to ensure that no relevant articles would be missed. Adding another term, such as ‘immunohistochemistry’, might have made the search more specific, but would also have increased the risk of eliminating relevant titles. After removal of duplicates, titles and abstracts were screened by two researchers independently (B.K. and S.B.) based on predefined inclusion and exclusion criteria (see Table S2). Remaining articles were read in full, and a further selection was made based on the relevance of these full texts. Discrepancies between the two researchers were discussed and resolved by consensus.

### Inclusion Criteria

Studies were included if they evaluated interassay, interobserver and/or interlaboratory concordance of at least two PD‐L1 IHC assays and/or LDTs used on tissue from NSCLC patients in clinical practice. Studies examining interobserver and/or interlaboratory concordance in only one assay were also included. In order for studies to qualify, determination of PD‐L1 expression had to be performed on histological tissue from NSCLC patients and appropriate scoring methods had to be used (i.e. assessment of membranous staining of tumour cells by at least one pathologist). Since PD‐L1 IHC was validated in histological specimens, studies examining cytological specimens only were excluded. Studies that did not perform adequate statistical analysis to compare assays (i.e. overall percentage of agreement should at least be given) were also excluded. Only articles written in English and containing original published data were eligible for inclusion.

### Quality Assessment

Methodological quality of all articles remaining after full text reading were appraised by using a revised form of the Quality Assessment of Diagnostic Accuracy Studies 2 (QUADAS‐2) tool for assessing risk of bias.^26^ Originally, this tool consists of four domains, i.e. patient selection, index test, reference standard and flow and timing. As individual PD‐L1 IHC assays were not compared to a reference standard in the included studies, but rather to each other, the reference standard domain was excluded from the QUADAS‐2 tool for this review. Instead, another domain was added based on the Quality in Prognosis Studies (QUIPS) tool, i.e. statistical analysis and reporting.^27^ Risk of bias was scored as low, moderate or high for each domain of the revised QUADAS‐2 tool and points were awarded accordingly (1 point for low risk of bias, 0.5 points for moderate risk of bias and 0 points for high risk of bias). Based on the sum of the scores given to each individual domain, overall scores of low, moderate or high risk of bias were awarded to studies using the following scoring system: low risk of bias for studies with ≥3.5 points, moderate risk of bias for studies with ≥2.5 and <3.5 points and high risk of bias for studies with <2.5 points. Appraisal of methodological quality was performed independently by two researchers (B.K. and S.B.) and differences were resolved through discussion. Studies with high risk of bias were excluded from data extraction and further analysis.

### Data Extraction And Synthesis Of Results

The following data were extracted from each study included after appraisal of methodological quality: first author’s name, year of publication, sample size, type of cancer of included patients, type of material used for PD‐L1 testing, type of standardised assay and/or LDT used for PD‐L1 testing, scoring method, cut‐off values, number of observers scoring PD‐L1, type of statistical analysis and results from comparison between assays, observers and/or laboratories. This review focuses on concordance of tumour cell (TC) staining and scoring, as treatment decisions for NSCLC patients are based on scoring of PD‐L1 expression on TCs in clinical practice. However, as scoring of PD‐L1 expression on immune cells (IC) could become relevant to clinical practice in the future, we also extracted data on concordance of IC staining and scoring and included this as Data S1 and Table S8. Due to heterogeneity between included studies, such as differences in antibodies tested, number of pathologists scoring and statistical methods applied, results could not be quantitatively pooled and a meta‐analysis could not be performed.

## Results

### Systematic Search And Study Selection

**Figure 1:**
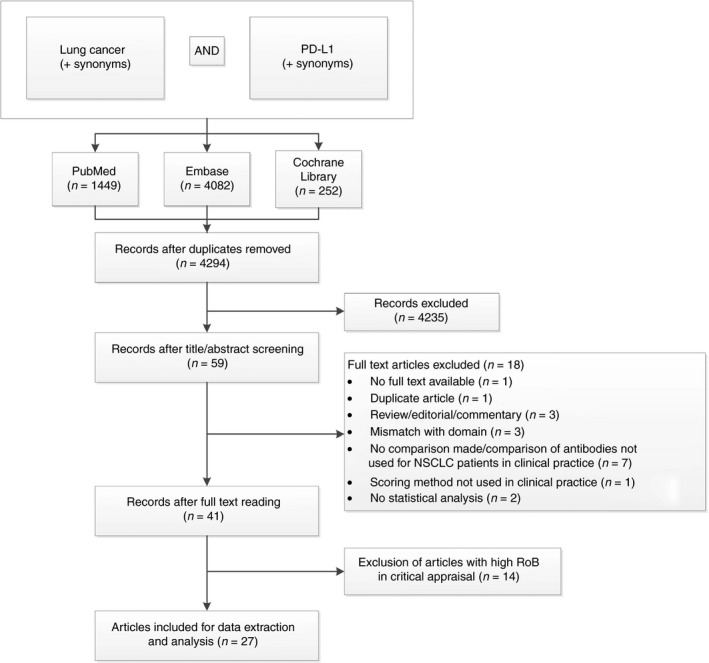
Flowchart of study selection process (date of search: 27 June 2018). PD‐L1, programmed cell death‐ligand 1; RoB, risk of bias.

The search in PubMed, Embase and Cochrane Library yielded 4294 unique hits after removal of duplicates (see Figure 1). Fifty‐nine records remained after screening of titles and abstracts. Of these, one full text was unavailable. Therefore, 58 full text articles were evaluated in detail, of which 41 articles met the inclusion criteria. All selected articles studied interassay, interobserver and/or interlaboratory concordance of at least one PD‐L1 IHC assay, using material from NSCLC patients. Most studies included multiple subtypes of NSCLC, with adenocarcinoma and squamous cell carcinoma being studied most frequently. Some studies also included patients with other types of lung cancer, such as small cell lung cancer (SCLC)^28^, ^29^ and mesothelioma.^30^ Sample sizes ranged from 15 to 713 tissue specimens. All studies used statistical analysis to measure concordance. The statistical methods used, however, varied. The kappa statistic (κ) was used most, but some studies used intraclass correlation coefficient (ICC), Pearson’s/Spearman’s correlation or calculation of percentage agreement.

### Quality Assessment

The 41 articles selected through full text reading were critically appraised on methodological quality. Based on scoring with the revised QUADAS‐2 tool, studies ranged from low to high risk of bias (see Table S3). Studies with high risk of bias were often unclear concerning their method of patient selection and reasons for patient exclusion, about blinding of pathologists for each other’s results and for the specific antibody used, about the use of staining platform and staining protocol, or about the scoring method used. Also, some studies did not provide sufficient information on the use of statistical methods or did not present all data, prohibiting assessment of adequacy of analytical strategy. Five studies were judged as having low risk of bias,^29^, ^31^, ^32^, ^33^, ^34^ 22 studies as having moderate risk of bias^22^, ^23^, ^24^, ^25^, ^28^, ^30^, ^35^, ^36^, ^37^, ^38^, ^39^, ^40^, ^41^, ^42^, ^43^, ^44^, ^45^, ^46^, ^47^, ^48^, ^49^, ^50^ and 14 studies as having high risk of bias.^51^, ^52^, ^53^, ^54^, ^55^, ^56^, ^57^, ^58^, ^59^, ^60^, ^61^, ^62^, ^63^, ^64^ The 14 studies with high risk of bias were excluded, which left 27 articles for data extraction and further analysis. An overview of study characteristics of all included studies can be found in Table S4.

### Interassay Concordance

Of the 27 included articles, 22 reported on interassay concordance of TC staining between PD‐L1 IHC assays. A summary of results from all 22 studies can be found in Table 1, while a more detailed presentation of results from each study can be found in Table S5. Many studies compared the standardised assays 22C3, 28‐8, SP263 and SP142. Overall, moderate to strong concordance was seen between 22C3, 28‐8 and SP263^22^, ^23^, ^28^, ^31^, ^32^, ^35^, ^38^, ^43^, ^48^ and lower concordance between SP142 and the other assays.^22^, ^24^, ^28^, ^31^, ^32^, ^35^, ^38^, ^39^, ^48^, ^50^ Concordance was often highest between assays 22C3 and 28‐8,^22^, ^30^, ^32^, ^35^, ^48^ such as demonstrated by Brunnström *et al*.,^32^ who found a weighted к value of 0.891 (0.82–0.96) for comparison of these two assays. Two studies by Scheel *et al*.^25^, ^46^ showed somewhat lower concordance values between 22C3, 28‐8 and SP263 than the other studies, but these results may have been affected by interobserver variation and by the low sample size in both studies. Several studies described a higher proportion of stained TCs with use of antibody SP263 when compared to antibody 22C3 and/or 28‐8.^25^, ^28^, ^35^, ^44^, ^46^, ^48^ According to Munari *et al*.,^44^ this difference in staining led to a significantly lower proportion of positive cases with assay 22C3 compared to assay SP263 for both the 1 and 50% cut‐offs. Similarly, other studies also assessed concordance with deployment of clinically relevant cut‐offs. Some of these studies showed diminished concordance rates when cut‐offs were used. Hendry *et al*.^28^ showed only moderate agreement between 22C3, 28‐8 and SP263 when cut‐offs were used (Cohen’s κ range = 0.433–0.631), while good agreement was found for PD‐L1 expression on a continuous scale (ICC range = 0.726–0.812). In the Blueprint Phase 1 study, agreement with the reference assay ranged from 86.8% to 94.7% for comparisons between antibody 22C3, 28‐8 and SP263 when different cut‐offs were used, meaning that in some cases almost 15% of patients in the study would not have been assigned a treatment if an alternative to the reference assay had been used.^22^ Other studies showed lower agreement for the 1% than for the 50% cut‐off.^35^, ^38^, ^43^ Two studies showed good concordance between assays for any cut‐off used,^23^, ^30^ but these studies only calculated percentage agreement, which may overestimate true agreement.^65^, ^66^

**Table 1:** Summary of results from studies assessing interassay concordance of TC staining

Type of test	Comparison	Interassay concordance
Standardised assays	22C3, 28‐8 and SP263	Moderate to high concordance for all comparisons^22^, ^23^, ^28^, ^31^, ^32^, ^35^, ^38^, ^43^, ^48^ Highest concordance between 22C3 and 28‐8^22^, ^30^, ^32^, ^35^, ^48^ Lower concordance rates with use of cut‐offs^22^, ^28^, ^44^, especially using the 1% cut‐off^35^, ^38^, ^43^
	SP142 versus all other assays	Lower concordance levels compared to comparisons between all other assays^22^, ^24^, ^28^, ^31^, ^32^, ^35^, ^38^, ^39^, ^48^, ^50^
LDTs	Various LDTs versus standardised assays	High concordance for some LDTs, only if appropriate protocol used^31^, ^32^, ^47^
	22C3 LDT versus 22C3 standardised assay	High correlation^28^, ^40^, ^41^, ^44^ In some studies higher correlation than between two different standardised assays^28^, ^44^
	E1L3N versus all standardised assays	High concordance between E1L3N and 22C3, 28‐8 and SP263^24^, ^37^, ^47^ Lower concordance between E1L3N and SP142^24^, ^42^

LDT, Laboratory‐developed test; TC, Tumour cell.

Comparisons of TC staining were also made between the 22C3, 28‐8, SP263 and SP142 antibodies being used with their standard protocols and being used in LDTs. A study by Adam *et al*.^31^ demonstrated that 14 of 27 LDTs were concordant (defined as weighted κ value ≥0.75) with one of the pre‐specified reference assays. The lowest κ value was seen for the SP142 LDT compared to the SP263 reference assay (weighted κ = 0.38). Two studies by Ilie *et al*.^40^, ^41^ showed high correlation between two different 22C3 LDTs and the 22C3 standardised assay. Another study also showed excellent agreement between the 22C3 standardised assay and LDT, with an ICC of 0.921 and Cohen’s κ of 0.897 for the 50% cut‐off.^28^ In this study, discrepancies were actually much greater between two different antibodies used on the same platform (22C3 and 28‐8) than between the same antibody (22C3) used on different platforms. A similar finding was reported by Munari *et al*.^44^ Other studies also compared one or more of the aforementioned standardised assays with antibody E1L3N, which is used as an LDT by some laboratories in clinical practice. Good correlation was seen between E1L3N and assays SP263, 28‐8 and 22C3,^24^, ^37^, ^47^ while comparison with SP142 again showed lower concordance values.^24^, ^42^ One study^36^ showed higher sensitivity in staining of PD‐L1 using 28‐8 compared to E1L3N. Finally, in a study by Soo *et al*.,^47^ which used the SP142 antibody as an LDT, changes in the SP142 protocol led to a higher intensity of staining compared to the original protocol, demonstrating how the IHC protocol can influence the apparent level of PD‐L1 expression.

### Interobserver Concordance

Sixteen of the 27 included studies examined interobserver concordance (see Table 2; Table S6). All these studies assessed concordance between pathologists scoring TC staining and most found moderate to almost perfect agreement for all assays.^23^, ^24^, ^29^, ^32^, ^33^, ^34^, ^35^, ^37^, ^39^, ^40^, ^45^, ^48^, ^49^ Only Scheel *et al*.^25^ found somewhat lower concordance values for E1L3N and SP142 LDTs and 22C3, 28‐8, SP263 and SP142 standardised assays when a scoring system applying five cut‐offs was used (Light’s κ range = 0.47–0.50). However, the sample size in this study was very small (*n* = 15), and classifying the cases by the dichotomous cut‐off criteria included in the scoring system resulted in higher concordance levels for all antibodies (Light’s κ range = 0.59–0.80). Other studies also assessed interobserver concordance for multiple cut‐offs, and many found concordance levels to be lower for the 1% cut‐off compared to the 50% cut‐off^23^, ^24^, ^29^, ^32^, ^35^, ^43^, ^48^ and the 5%, 10% or 25% cut‐off.^23^, ^32^, ^48^ The Blueprint Phase 2 study also assessed the 80% cut‐off and found interpathologist agreement to be slightly diminished for this cut‐off compared to the 5%, 10%, 25% and 50% cut‐offs.^48^ A study by Cooper *et al*.^33^ actually reported lower concordance levels for the 50% cut‐off than for the 1% cut‐off for assay 22C3 (overall percentage agreement (OPA) 81.9% and κ = 0.58 versus OPA 84.2% and κ = 0.69, respectively). However, this study reported prevalence bias to have influenced the κ magnitude for the 50% cut‐off. These results therefore have to be interpreted with caution.

**Table 2:** Summary of results from studies assessing interobserver concordance of TC scoring

Type of test	Overall	Use of cut‐offs
Standardised assays	Good concordance for all standardised assays^23^, ^24^, ^29^, ^32^, ^33^, ^34^, ^35^, ^37^, ^39^, ^40^, ^43^, ^44^, ^45^, ^48^, ^49^ One study showing only moderate agreement^25^	Lower concordance levels for 1% cut‐off compared to 50% cut‐off^23^, ^24^, ^29^, ^32^, ^35^, ^43^, ^48^ Lower concordance levels for 1% cut‐off compared to 5%, 10% and 25% cut‐offs^23^, ^32^, ^48^ Lower concordance levels for 80% cut‐off compared to other cut‐offs^48^
LDTs	Good concordance for various LDTs^24^, ^29^, ^32^, ^37^, ^40^, ^44^, ^45^	Lower concordance levels for 1% cut‐off compared to other cut‐offs^24^, ^29^, ^32^

LDT, Laboratory‐developed test; TC, Tumour cell.

### Interlaboratory Concordance

Interlaboratory concordance of TC staining was assessed by five of the 27 included studies (see Table 3; Table S7). Two of these^34^, ^49^ assessed only one antibody (22C3 and SP142, respectively). Both studies found high interlaboratory agreement. Adam *et al*.,^31^ who assessed interlaboratory concordance for 22C3, 28‐8 and SP263, found very high agreement between participating centres for each of these assays. Marchetti *et al*.^43^ found similar results for the assays 22C3 and SP263. Scheel *et al*.^46^ assessed interlaboratory concordance for standardised assays 22C3, 28‐8, SP263 and SP142 and for 22C3, 28‐8, SP263 and E1L3N used in LDTs, performed in 10 different sites. Concordance values ranged from Light’s κ = 0.63–0.69 for the standardised assays when five cut‐offs were used. κ was 0.49 for all the LDTs grouped together. When only a 1% and 50% cut‐off were used, concordance values improved to κ = 0.73–0.89 for the standardised assays and κ = 0.5 for the LDTs.

**Table 3:** Summary of results from studies assessing interlaboratory concordance of TC scoring

Type of test	Interlaboratory concordance
Standardised assays	22C3: substantial to near‐perfect concordance^31^, ^34^, ^43^, ^46^ 28‐8: substantial to near‐perfect concordance^31^, ^46^ SP263: substantial to near‐perfect concordance^31^, ^43^, ^46^ SP142: high intersite percentage agreement^49^
LDTs	Only moderate concordance levels compared to standardised assays^46^

LDT, Laboratory‐developed test; TC, Tumour cell.

### Concordance Of Immune Cell Staining And Scoring

A short analysis of concordance of IC staining and scoring can be found as Data S1 and Table S8.

## Discussion

Ever since the approval of PD‐1 and PD‐L1 inhibitors as treatment options for patients with advanced NSCLC, various studies have been published assessing the comparability of different PD‐L1 IHC assays. In this systematic review, interassay, interobserver and interlaboratory concordance of these PD‐L1 IHC assays and LDTs were investigated by reviewing all currently available literature.

Overall, interassay agreement of TC staining is high between standardised assays 22C3, 28‐8 and SP263, while assay SP142 frequently shows lower staining of TCs. Agreement between LDTs and their reference assay may also be high, depending on the protocol that is used, with some studies even showing greater agreement between LDTs and their reference assays than between different standardised assays.^28^, ^44^ These data seem to suggest that the assays 22C3, 28‐8 and SP263 and properly validated LDTs could be used interchangeably on histological specimens of NSCLC patients. However, some studies have shown lower concordance levels with the use of clinically relevant cut‐offs.^22^, ^28^, ^44^ The 1% cut‐off especially may lead to higher disagreement compared to the 50% cut‐off,^35^, ^38^, ^43^ although this could perhaps be attributed to lower interrater agreement levels at this cut‐off.^43^ Based on the lower concordance levels found when using various cut‐offs, it would be too premature to draw the conclusion that assays and LDTs can be used interchangeably without any consequences. Notably, in a recent meta‐analysis of diagnostic accuracy of PD‐L1 IHC assays, Torlakovic *et al*.^67^ demonstrated that none of the standardised PD‐L1 assays could be deemed as interchangeable, when interchangeability is defined as achieving ≥90% sensitivity and specificity for both the 1 and 50% cut‐offs. Because discordance may exist between assays at clinically relevant cut‐offs, simply interchanging one assay with another may potentially lead to patients being wrongfully denied valuable treatment options in clinical practice.

Assessment of interobserver concordance of TC scoring showed that agreement between pathologists is moderate to high for all assays and LDTs. Markedly, agreement is often found to be lowest for the 1% cut‐off compared to other cut‐offs. This is problematic, especially now that the European Medicines Agency (EMA) has only approved durvalumab as consolidation treatment in stage III NSCLC patients whose tumours show PD‐L1 expression of ≥1%.^12^ One could question if the use of this cut‐off provides results that are reliable enough to aid clinicians in making treatment decisions. Agreement is likely to be higher between more experienced pathologists,^43^ yet still leaves room for improvement. One study assessing training of already experienced pathologists showed no or only little improvement of interobserver agreement.^33^ This study, however, employed a 1‐h training session consisting of a presentation only. Alternative training initiatives, preferably including a more practical element during which trainees have to perform PD‐L1 scoring on multiple specimens, might prove to be more effective. A recent study assessing interpathologist concordance of PD‐L1 scoring using real‐world data showed that training for PD‐L1 scoring and experience in routine pathology practice correlated with higher concordance.^68^ The effect of training on interobserver concordance should thus be studied more extensively. Other solutions also deserve more attention, especially the use of digital image analysis for PD‐L1 scoring, as this has been shown to reduce interobserver variability.^69^

Finally, we assessed concordance of PD‐L1 IHC assays and LDTs between laboratories. Only a limited number of studies assessed this type of concordance, especially compared to the large number of studies assessing interassay and/or interobserver concordance. Most of the studies assessing interlaboratory concordance found high agreement for all standardised assays, while one study found lower agreement for LDTs.^46^ However, not all these studies used the right study protocol and the right outcome measure to properly assess interlaboratory concordance. Two studies^34^, ^49^ used percentage of agreement as outcome measure, which does not account for random agreement and may thus overestimate true agreement.^65^, ^66^ Two other studies^43^, ^46^ used study designs that did not allow for separate analysis of interobserver and interlaboratory concordance. Moreover, none of the study designs allowed for assessment of the influence of pre‐analytical variables on PD‐L1 immunostaining, while in clinical practice pre‐analytical processing of samples may actually differ considerably between laboratories and may influence IHC staining results.^70^, ^71^, ^72^ Therefore, studies assessing interlaboratory variation in PD‐L1 expression are needed, using real‐world data and thereby taking into account these possible differences in pre‐analytical variables in clinical practice.

This systematic review has some limitations. Most importantly, there is significant heterogeneity between the studies included, especially in the choice of antibodies tested and the statistical methods used to analyse concordance. This prohibits pooling of data and complicates proper comparison of results between studies. Most studies, however, used similar samples for PD‐L1 testing, i.e. formalin‐fixed paraffin‐embedded material from tumour resections or biopsies from NSCLC patients. This supports comparability between studies. Conversely, this also provides a disadvantage: it only allows for comparison of PD‐L1 IHC assays and LDTs in histology, while in clinical practice PD‐L1 immunostaining is frequently performed on cytological specimens. Comparison of PD‐L1 IHC assays and LDTs in cytological NSCLC specimens falls beyond the scope of this review, but would be worth evaluation in a separate study. Finally, many of the included studies were not of high methodological quality, with only five studies being judged as having low risk of bias. Excluding the studies with the highest level of risk of bias, however, has improved the overall quality of this review.

To conclude, this systematic review has shown that interassay concordance of TC staining is generally high between the standardised assays 22C3, 28‐8 and SP263 and properly developed and validated LDTs. Nevertheless, the use of clinically relevant cut‐offs may lead to lower levels of interassay concordance, indicating that these assays and LDTs cannot simply be interchanged. Interobserver agreement, moreover, is generally high for all assays and LDTs, but decreases with use of the 1% cut‐off. Lastly, interlaboratory concordance seems to be high for standardised assays and moderate for LDTs, but has not been studied sufficiently to draw definitive conclusions. Studies using real‐world clinical pathology data are necessary to assess whether use of different PD‐L1 IHC assays and LDTs, scoring by different pathologists and use of different pre‐analytical variables actually lead to differences in PD‐L1 positivity between laboratories in clinical practice.

## Conflicts of interest

B. K. and S. W. received research grants from AstraZeneca, MSD and Roche Diagnostics. S. W. also received research grants from BMS, NextCure and Pfizer. M. H. received research grants from AstraZeneca, BMS, MSD, Pfizer, Roche and Roche Diagnostics. S. B. declares no conflicts of interest. None of the grant suppliers were involved in the study design, collection, analysis and interpretation of data, writing of the manuscript or the decision to submit the manuscript for publication.

## Supporting information


**Table S1** **.** Search syntax in PubMed, Embase and Cochrane Library.
**Table S2** **.** Inclusion and exclusion criteria.
**Table S3** **.** Quality assessment of included studies.
**Table S4** **.** Study characteristics of all studies included for data extraction and analysis.
**Table S5** **.** Results from studies assessing inter‐assay concordance of TC staining.
**Table S6** **.** Results from studies assessing inter‐observer concordance of TC scoring.
**Table S7** **.** Results from studies assessing inter‐laboratory concordance of TC staining.
**Table S8** **.** Results from studies assessing inter‐assay and/or inter‐observer concordance of IC staining/scoring.
**Data S1** **.** Supplementary results: concordance of IC staining and scoring.
