# Supplementary material for: Comparability of PD‐L1 immunohistochemistry assays for non‐small‐cell lung cancer: a systematic review
Source: Histopathology. 2020 Mar 24;76(6):793–802. doi: 10.1111/his.14040 (PMC7318295; doi:10.1111/his.14040)
Supplement: Supplementary file 1 — Table S1 . Search syntax in PubMed, Embase and Cochrane Library. Table S2 . Inclusion and exclusion criteria. Table S3 . Quality assessment of included studies. Table S4 . Study characteristics of all studies included for data extraction and analysis. Table S5 . Results from studies assessing inter‐assay concordance of TC staining. Table S6 . Results from studies assessing inter‐observer concordance of TC scoring. Table S7 . Results from studies assessing inter‐laboratory concordance of TC staining. Table S8 . Results from studies assessing inter‐assay and/or inter‐observer concordance of IC staining/scoring. Data S1 . Supplementary results: concordance of IC staining and scoring. [file HIS-76-793-s001.docx]

**SUPPORTING INFORMATION**

**Comparability of PD-L1 immunohistochemistry assays for non-small cell lung cancer: a systematic review**

B.M. Koomen, S.K. Badrising, M.M. van den Heuvel, S.M. Willems

| **Supplementary table 1.** Search syntax in PubMed, Embase and Cochrane Library  (date of search: June 27, 2018). | | |
| --- | --- | --- |
| **Database** | **Search syntax** | **Hits** |
| PubMed | (((lung[Title/Abstract] OR lungs[Title/Abstract] OR pulmonary[Title/Abstract]) AND (cancer*[Title/Abstract] OR carcinom*[Title/Abstract] OR neoplasm*[Title/Abstract] OR tumour*[Title/Abstract] OR tumour*[Title/Abstract])) OR lung adenocarcinom*[Title/Abstract] OR pulmonary adenocarcinom*[Title/Abstract] OR NSCLC[Title/Abstract] OR carcinoma, non small cell lung[MeSH Terms])  AND  (PD-L1[Title/Abstract] OR PDL1[Title/Abstract] OR "Programmed death-ligand 1"[Title/Abstract] OR “Programmed cell death-ligand 1”[Title/Abstract] OR B7-H1[Title/Abstract] OR B7H1[Title/Abstract] OR CD274[Title/Abstract] OR B7-H1 antigen[MeSH Terms]) | 1449 |
| Embase | (((lung:ti,ab OR lungs:ti,ab OR pulmonary:ti,ab) AND (cancer*:ti,ab OR carcinom*:ti,ab OR tumour*:ti,ab OR tumour*:ti,ab OR neoplasm*:ti,ab)) OR 'lung adenocarcinom*':ti,ab OR 'pulmonary adenocarcinom*':ti,ab OR nsclc:ti,ab OR 'non small cell lung cancer'/exp)  AND  ('pd l1':ti,ab OR pdl1:ti,ab OR 'programmed cell death-ligand 1':ti,ab OR 'programmed death-ligand 1':ti,ab OR 'b7 h1':ti,ab OR b7h1:ti,ab OR cd274:ti,ab OR 'programmed death 1 ligand 1'/exp)  AND  [embase]/lim | 4082 |
| Cochrane Library | (((lung:ti,ab OR lungs:ti,ab OR pulmonary:ti,ab) AND (cancer*:ti,ab OR carcinom*:ti,ab OR tumour*:ti,ab OR tumour*:ti,ab OR neoplasm*:ti,ab)) OR "lung adenocarcinom*":ti,ab OR "pulmonary adenocarcinom*":ti,ab OR nsclc:ti,ab)  AND  (PD-L1:ti,ab OR PDL1:ti,ab OR "Programmed death-ligand 1":ti,ab OR "Programmed cell death-ligand 1":ti,ab OR B7-H1:ti,ab OR B7H1:ti,ab OR CD274:ti,ab) | 252 |

| **Supplementary table 2.** Inclusion and exclusion criteria. | |
| --- | --- |
| **Inclusion criteria** | |
| Population | Patients with non-small cell lung cancer (any type). |
| Study design | - Comparison of at least two commercially available standardised assays and/or LDTs for detection of PD-L1 expression in NSCLC patients; - Studies examining only one assay may be included when inter-observer concordance and/or inter-laboratory concordance is assessed. |
| Outcome | - Inter-assay and/or inter-observer and/or inter-laboratory concordance of PD-L1 staining/scoring are assessed; - Proper statistical analysis is performed (overall percentage of agreement should at least be given). |
| **Exclusion criteria** | |
|  | - Comparison of assays/LDTs only used for evaluating PD-L1 expression in types of cancer other than NSCLC; - Use of cytological material only; - Use of scoring method not employed in clinical practice; - Language other than English; - Duplicate articles containing all/some of original publicised data; - Reviews, conference abstracts, case reports, editorials, book chapters, presentations. |

| **Supplementary table 3.** Quality assessment of included studies. | | | | | | |
| --- | --- | --- | --- | --- | --- | --- |
| **First author** | **Year** | **Patient selection** | **Test(s)** | **Flow and timing** | **Statistical analysis and reporting** | **Risk of Bias (RoB)** |
| Adam^31^ | 2018 | 🌓 | 🌑 | 🌑 | 🌑 | Low |
| Brunnström^32^ | 2017 | 🌑 | 🌑 | 🌓 | 🌑 | Low |
| Chan^35^ | 2018 | 🌑 | 🌓 | 🌓 | 🌑 | Moderate |
| Cogswell^36^ | 2017 | 🌕 | 🌓 | 🌑 | 🌑 | Moderate |
| Conde^37^ | 2018 | 🌑 | 🌓 | 🌓 | 🌑 | Moderate |
| Cooper^33^ | 2017 | 🌑 | 🌑 | 🌑 | 🌓 | Low |
| Erber^51^ | 2017 | 🌓 | 🌓 | 🌓 | 🌓 | High |
| Fujimoto^38^ | 2017 | 🌓 | 🌑 | 🌓 | 🌑 | Moderate |
| Hendry^28^ | 2017 | 🌓 | 🌓 | 🌓 | 🌑 | Moderate |
| Hirsch^22^ | 2016 | 🌓 | 🌑 | 🌓 | 🌑 | Moderate |
| Ilie^39^ | 2016 | 🌓 | 🌓 | 🌓 | 🌑 | Moderate |
| Ilie^40^ | 2017 | 🌓 | 🌑 | 🌑 | 🌓 | Moderate |
| Ilie^41^ | 2018 | 🌓 | 🌑 | 🌑 | 🌓 | Moderate |
| Keller^42^ | 2018 | 🌓 | 🌓 | 🌓 | 🌑 | Moderate |
| Kim^52^ | 2017 | 🌓 | 🌓 | 🌓 | 🌓 | High |
| Krawczyk^53^ | 2017 | 🌓 | 🌕 | 🌓 | 🌓 | High |
| Marchetti^43^ | 2017 | 🌑 | 🌓 | 🌓 | 🌑 | Moderate |
| McLaughlin^54^ | 2016 | 🌓 | 🌕 | 🌓 | 🌑 | High |
| Munari^44^ | 2018 | 🌑 | 🌓 | 🌓 | 🌑 | Moderate |
| Neuman^55^ | 2016 | 🌑 | 🌓 | 🌕 | 🌓 | High |
| Pang^56^ | 2018 | 🌓 | 🌓 | 🌓 | 🌓 | High |
| Parra^57^ | 2018 | 🌕 | 🌓 | 🌓 | 🌓 | High |
| Paulsen^58^ | 2017 | 🌓 | 🌕 | 🌓 | 🌓 | High |
| Phillips^59^ | 2015 | 🌕 | 🌓 | 🌕 | 🌑 | High |
| Ratcliffe^23^ | 2017 | 🌓 | 🌓 | 🌑 | 🌑 | Moderate |
| Rebelatto^60^ | 2016 | 🌕 | 🌓 | 🌓 | 🌓 | High |
| Rehman^45^ | 2017 | 🌓 | 🌓 | 🌓 | 🌑 | Moderate |
| Rimm^24^ | 2017 | 🌓 | 🌓 | 🌓 | 🌑 | Moderate |
| Roach^34^ | 2016 | 🌑 | 🌑 | 🌓 | 🌑 | Low |
| Roge^61^ | 2017 | 🌓 | 🌓 | 🌓 | 🌓 | High |
| Russell-Goldman^29^ | 2018 | 🌑 | 🌑 | 🌓 | 🌑 | Low |
| Scheel^25^ | 2016 | 🌓 | 🌑 | 🌓 | 🌑 | Moderate |
| Scheel^46^ | 2018 | 🌓 | 🌓 | 🌓 | 🌑 | Moderate |
| Sheffield^62^ | 2016 | 🌓 | 🌓 | 🌓 | 🌓 | High |
| Skov^30^ | 2017 | 🌓 | 🌑 | 🌓 | 🌑 | Moderate |
| Smith^63^ | 2016 | 🌓 | 🌓 | 🌓 | 🌓 | High |
| Soo^47^ | 2018 | 🌓 | 🌓 | 🌓 | 🌑 | Moderate |
| Tsao^48^ | 2018 | 🌓 | 🌓 | 🌓 | 🌑 | Moderate |
| Tseng^64^ | 2018 | 🌓 | 🌓 | 🌓 | 🌓 | High |
| Vennapusa^49^ | 2018 | 🌕 | 🌑 | 🌑 | 🌓 | Moderate |
| Xu^50^ | 2017 | 🌓 | 🌓 | 🌓 | 🌑 | Moderate |
| Legend: 🌑 = low RoB (1 point), 🌓 = moderate RoB (0.5 points), 🌕 = high RoB (0 points). Scoring system: ≥3.5 points = low RoB; ≥2.5 and <3.5 points = moderate RoB; <2.5 points = high RoB.  For references see main manuscript reference list. | | | | | | |

| **Supplementary table 4.** Study characteristics of all studies included for data extraction and analysis. | | | | | | | |
| --- | --- | --- | --- | --- | --- | --- | --- |
| **First author** | **Year** | **Samples analysed** | **Antibodies compared** | **Scoring method** | **Cut-offs** | **Number of observers** | **Statistical methods** |
| Adam^31^ | 2018 | 41 FFPE resection samples (AC, SCC) | Standardised assays: 22C3, 28-8, SP263  LDTs: 22C3, 28-8, SP263, SP142, E1L3N | Percentage of TC staining (membranous staining of any intensity) | 1%, 50% | 1 | - Inter-assay concordance: weighted κ, OPA - Inter-laboratory concordance: weighted κ |
| Brunnström^32^ | 2017 | TMAs from 55 FFPE tissue blocks (AC, SCC, LCNEC, LCC) | Standardised assays: 22C3, 28-8, SP263, SP142  LDTs: 28-8 | Percentage of TC staining (membranous staining of any intensity) | 1%, 5%, 25%, 50% | 7 | - Inter-assay concordance: weighted κ - Inter-observer concordance: weighted κ |
| Chan^35^ | 2018 | TMAs from 713 FFPE resection samples (AC, SCC, ASC, LCC, SC, LELC) | Standardised assays: 22C3, 28-8, SP263, SP142 | Percentage of TC staining (membranous staining of any intensity) | 1%, 50% | 2 | - Inter-assay concordance: scatter plots with Pearson’s *R*^2^ - Inter-observer concordance: ICC, Cohen’s κ |
| Cogswell^36^ | 2017 | 40 tissue samples (20 from NSCLC, 20 from melanoma) | Standardised assays: 28-8  LDTs: E1L3N | Percentage of TC staining (partial or complete membranous staining) | Not reported | 3 | Determining sensitivity in staining of PD-L1 |
| Conde^37^ | 2018 | - Discovery cohort: 40 FFPE resection samples (SCC). - Validation cohort: 29 FFPE resection samples (SCC). | Standardised assays: SP263, SP142  LDTs: E1L3N | Percentage of TC staining (membranous and/or cytoplasmic staining) | 1%, 5%, 10%, 25%, 50% | 2 | - Inter-assay concordance: Pearson’s correlation coefficient - Inter-observer concordance: ICC, Fleiss’ κ |
| Cooper^33^ | 2017 | TMAs from 108 FFPE resection samples (NSCLC) | Standardised assays: 22C3 | Percentage of TC staining (membranous staining of any intensity) | 1%, 50% | 10 | Inter-observer concordance: OPA, NPA, PPA, Cohen’s κ |
| Fujimoto^38^ | 2017 | 40 FFPE tumour samples (AC, SCC, other) | Standardised assays: 22C3, 28-8, SP263, SP142 | Percentage of TC staining (membranous staining of any intensity) | 1%, 50% | 4 | Inter-assay concordance: weighted κ |
| Hendry^28^ | 2017 | TMAs from 423 resections (lung malignancies) | Standardised assays: 22C3, 28-8, SP263, SP142  LDTs: 22C3 | Percentage of TC staining (membranous staining of any intensity) | 1%, 25%, 50%. | 1 | Inter-assay concordance: ICC, OPA, NPA, PPA, Cohen's κ |
| Hirsch^22^ | 2017 | 38 FFPE resection and biopsy samples (NSCLC) | Standardised assays: 22C3, 28-8, SP263, SP142 | Percentage of TC staining (partial or complete membranous staining) | 1%, 25%. | 3 | Inter-assay concordance: scatter plots, regression analysis. |
| Ilie^39^ | 2016 | 56 FFPE resection samples (basaloid SCC) | Standardised assays: SP263, SP142  LDTs: 28-8 | Percentage of TC staining (unclear if only membranous staining or also cytoplasmic) | - SP142: 1%, 5%, 50%; - SP263: 25%; - 28-8: 1%, 5%, 10% | 3 | - Inter-assay concordance: Spearman correlation coefficient, Cohen's κ - Inter-observer concordance: OPA, κ |
| Ilie^40^ | 2017 | 120 FFPE resection and biopsy samples (AC, SCC) | Standardised assays: 22C3  LDTs: 22C3 (2 protocols) | Percentage of TC staining (partial or complete membranous staining of any intensity) | 1%, 50% | 3 | - Inter-assay concordance: NPA, PPA, ICC. - Inter-observer concordance: κ |
| Ilie^41^ | 2018 | 37 FFPE bronchial biopsy samples (AC, SCC) | Standardised assays: 22C3  LDTs: 22C3 (2 protocols) | Percentage of TC staining (partial or complete membranous staining of any intensity) | 1%, 50% | 2 | Inter-assay concordance: ICC |
| Keller^42^ | 2018 | TMAs from 370 FFPE resection samples (SCC) | LDTs: SP142, E1L3N | Percentage of TC staining (membranous staining of any intensity) | 1%, 50% | 1 | Inter-assay concordance: Spearman's ρ correlation |
| Marchetti^43^ | 2017 | TMAs from 100 FFPE resection samples (AC) | Standardised assays: 22C3, SP263 | Percentage of TC staining (membranous staining of any intensity) | 1%, 50% | 4 pathologists/  4 centres | - Inter-assay concordance: Pearson's precision analysis, weighted κ/Light's κ - Inter-observer concordance: weighted κ/Fleiss' κ. - Inter-laboratory concordance: ICC |
| Munari^44^ | 2018 | TMAs from 198 FFPE resection samples (AC, SCC, other) | Standardised assays: 22C3, SP263  LDTs: 22C3 | Percentage of TC staining (membranous staining of any intensity) | 1%, 50% | 2 | - Inter-assay concordance: OPA, PPA, NPA, Cohen's κ - Inter-observer concordance: Cohen's κ |
| Ratcliffe^23^ | 2017 | 493 FFPE tissue samples (non-squamous carcinoma, SC, ASC) | Standardised assays: 22C3, 28-8, SP263 | Percentage of TC staining (membranous staining) | 1%, 10%, 25%, 50% | 2 | - Inter-assay concordance: Spearman correlation coefficient, OPA, NPA, PPA - Inter-observer concordance (determined in subset of 200 samples): OPA |
| Rehman^45^ | 2017 | 105 FFPE tissue samples (AC, SCC) | LDTs: SP142 | Percentage of TC staining (predominantly membranous staining at any intensity) | None | 5 | Inter-observer concordance: ICC |
| Rimm^24^ | 2017 | 90 resection samples (AC, SCC) | Standardised assays: 22C3, 28-8  LDTs: SP142, E1L3N | Percentage of TC staining (membranous and/or cytoplasmic staining) | 1%, 5%, 10%, 25%, 50% | 13 | - Inter-assay concordance: paired Wilcoxon signed-rank test, ICC. - Inter-observer concordance: ICC, Fleiss’ κ |
| Roach^34^ | 2016 | - Inter-observer concordance: 62 FFPE tissue samples (NSCLC) - Inter-laboratory concordance: 36 FFPE tissue samples (NSCLC) | Standardised assays: 22C3 | Percentage of TC staining (partial or complete membranous staining of intensity 1+ to 3+) | 50% | 3 pathologists/  3 centres | - Inter-observer concordance: OPA, NPA, PPA - Inter-laboratory concordance: OPA, NPA, PPA |
| Russell-Goldman^29^ | 2018 | 46 FFPE resection and biopsy samples (NSCLC) | LDTs: E1L3N | Percentage of TC staining (partial or complete membranous staining of any intensity) | 1%, 50% | 2 | Inter-observer concordance: Bland-Altman plots, ICC |
| Scheel^25^ | 2016 | - Training set: 15 FFPE tissue samples (AC, SCC) - Validation set: 15 FFPE tissue samples (AC, SCC) | Standardised assays: 22C3, 28-8, SP263, SP142  LDTs: SP142, E1L3N | Percentage of TC staining (partial or complete membranous staining) | 1%, 5%, 10%, 25%, 50% | 9 | - Inter-assay concordance: pairwise comparison of assays (percentage of concordance) - Inter-observer concordance: Cohen's κ and Light's κ |
| Scheel^46^ | 2018 | TMAs from 21 FFPE resection samples (NSCLC) | Standardised assays: 22C3, 28-8, SP263  LDTs: 22C3, 28-8, SP263, SP142, E1L3N, QR1 | Percentage of TC staining (unclear if only membranous staining or also cytoplasmic) | - 3-step score: 1%, 50% - 6-step score: 1%, 5%, 10%, 25%, 50% | 10 centres | - Inter-assay concordance: Light's κ - Inter-laboratory concordance: Cohen's κ of each local result compared with central reference score |
| Skov^30^ | 2017 | 87 FFPE resection and biopsy samples (AC, SCC, LCNEC, NSCLC NOS, mesothelioma, metastasis to lung) | Standardised assay: 22C3, 28-8 | Percentage of TC staining (partial or complete membranous staining of any intensity) | 50% | 1 | Inter-assay concordance: OPA, NPA, PPA, Pearson correlation coefficient |
| Soo^47^ | 2018 | 18 FFPE samples from resections, biopsies, pleural fluid and FNAs (NSCLC) | Standardised assays: 22C3, 28-8, SP263, SP142  LDTs: E1L3N | Percentage of TC staining (unclear if only membranous staining or also cytoplasmic) | Not reported | 3 | Inter-assay concordance: Spearman's rank test |
| Tsao^48^ | 2018 | 81 paraffin samples from resections, biopsies, lymph node excisions and cytological cell blocks (AC, SCC, NSCLC NOS, SCLC) | Standardised assays: 22C3, 28-8, SP263, SP142  Unclear: 73-10 | Percentage of TC staining (partial or complete membranous staining) | 1%, 5%, 10% 25%, 50%, 80% | 24 | - Inter-assay concordance: comparison of best-fit curves - Inter-observer concordance: ICC, Fleiss’ κ |
| Vennapusa^49^ | 2018 | - Inter-observer concordance: 80 FFPE resection and biopsy samples (NSCLC) - Inter-laboratory concordance: 28 FFPE resection and biopsy samples (NSCLC) | Standardised assays: SP142 | Percentage of TC staining (membranous staining of any intensity) | Combined scoring of TCs and ICs (TCs: 1%, 5%, 50%; ICs: 1%, 5%, 10%). | 3 pathologists/  3 centres | - Inter-observer concordance: OPA, NPA, PPA. - Inter-laboratory concordance: OPA, NPA, PPA |
| Xu^50^ | 2017 | 135 resection samples (AC, SCC, other) | Standardised assays: 22C3, SP142 | Percentage of TC staining (partial or complete membranous staining) | 1%, 5%, 50% | 2 | Inter-assay concordance: weighted κ, McNemar-Bowker test. |
| Abbreviations: AC=adenocarcinoma; ASC=adenosquamous carcinoma; FFPE=formalin-fixed paraffin-embedded; FNA=fine needle aspiration; IC=immune cell; ICC=intraclass correlation coefficient; κ=kappa; LCC=large cell carcinoma; LCNEC=large cell neuro-endocrine carcinoma; LDT=laboratory-developed test; LELC=lymphoepithelioma-like carcinoma; NPA=negative percent agreement; NSCLC NOS=non-small cell lung carcinoma not otherwise specified; OPA=overall percent agreement; PPA=positive percent agreement; SC=sarcomatoid carcinoma; SCC=squamous cell carcinoma; SCLC=small cell lung carcinoma; TC=tumour cell; TMA=tissue-microarray. References: see main manuscript reference list. | | | | | | | |

| **Supplementary table 5.** Results from studies assessing inter-assay concordance of TC staining. | | |
| --- | --- | --- |
| **First author** | **Year** | **Study results (inter-assay concordance)** |
| Adam^31^ | 2018 | - High concordance between assays 22C3, 28-8, and SP263 (к 0.71-0.89). - 14 of 27 LDTs (51.8%) concordant with one of the reference assays (к >0.75). |
| Brunnström^32^ | 2017 | - Inter-assay concordance range for comparison of all standardised assays and 28-8 LDT: к 0.45-0.91. Lowest values for comparison of SP142 with other assays (0.45-0.63). - Agreement between assays higher with 50% cut-off than with 1% cut-off. |
| Chan^35^ | 2018 | - High agreement between 22C3, 28-8 and SP263 (Pearson *R*^2^ 0.841-0.873). Lower correlation with SP142 (*R*^2^ ≈ 0.70). - Lower OPA (68.6-82%) at 1% cut-off compared to 50% cut-off (94.4-97.9%) |
| Cogswell^36^ | 2017 | - Higher sensitivity using 28-8 compared to E1L3N. - 28-8 assay more frequently detected PD-L1 positive TCs (22 vs. 6) compared with E1L3N. |
| Conde^37^ | 2018 | - Very good correlation between E1L3N and SP263 in both cohorts (ρ = 0.94 and ρ = 0.99). - Lower correlation between SP263 and SP142 (ρ = 0.88 and ρ = 0.87). |
| Fujimoto^38^ | 2017 | - Higher concordance between 22C3, 28-8 and SP263 (к 0.64-0.71) than between SP142 and other assays (к 0.39-0.55). - Higher agreement at 50% cut-off than at 1% cut-off. |
| Hendry^28^ | 2017 | - Good agreement between 22C3, 28-8, SP263 and SP142 on continuous scale (ICC 0.674). Higher when SP142 was excluded (ICC 0.755). - Moderate agreement between assays with use of clinical cut-offs (к 0.43). - Excellent agreement between 22C3 assay and 22C3 LDT (ICC 0.921, к 0.897 for cut-off 50%). |
| Hirsch^22^ | 2017 | - High correlation between 22C3, 28-8 and SP263, lower correlation for all comparisons including SP142. - Replacement of validated cut-off with any other cut-off reduced overall agreement for each assay. |
| Ilie^39^ | 2016 | - Poor correlation between SP142 and SP263 (к 0.362) or 28-8 (к 0.412). - Good correlation between SP263 and 28-8 (к 0.883). |
| Ilie^40^ | 2017 | High concordance between two 22C3 LDTs and 22C3 assay (ICC 98.7-99.9%). |
| Ilie^41^ | 2018 | High correlation between two 22C3 LDTs and 22C3 assay (ICC 0.999 and 1.000). |
| Keller^42^ | 2018 | Significant correlation of TPS between E1L3N and SP142 (*r* = 0.781; *P* < 0.001), although E1L3N showed higher sensitivity. |
| Marchetti^43^ | 2017 | - Correlation between 22C3 and SP263 0.89-0.97 for 4 participating centres. - 50% cut-off: к values for all centres 0.844-1. - 1% cut-off: к values for all centres 0.62-0.83. |
| Munari^44^ | 2018 | - OPA between 22C3 assay and SP263 77.3% (к 0.518) and 68.6% (к 0.390). 22C3 stained significantly lower proportion of cases than SP263. - OPA between 22C3 LDT and SP263 81.5% (к 0.624) and 76.1% (к 0.572). - OPA between 22C3 assay and 22C3 LDT 84.7% (к 0.595) and 80.3% (к 0.583). |
| Ratcliffe^23^ | 2017 | - High associations between assays 22C3, 28-8 and SP263 (Spearman correlations >0.9). - OPA of >90% between assays at multiple cut-offs. - NPA and PPA >85% for each comparison at different cut-offs. |
| Rimm^24^ | 2017 | - Only scores of 28-8 and E1L3N not statistically significantly different. - SP142 greatest magnitude of difference compared to the other 3 assays. - ICC based on average scores 0.81, which increased to 0.97 after SP142 exclusion. |
| Scheel^25^ | 2016 | - OPA 41%-72% for pairwise comparison of all standardised assays/LDTs. Highest concordance between 22C3 and 28-8. - SP142 stained lower proportions of TCs than 22C3 and 28-8, SP263 stained higher proportions of TCs. |
| Scheel^46^ | 2018 | - Similar proportions of TC staining for 22C3 and 28-8, fewer TCs stained with SP142, more TCs stained with SP263. - Similar staining patterns to 22C3 and 28-8 for 6 of 11 LDTs. - Substantial to near-perfect concordance between assay 22C3 and 28-8. - Moderate concordance between assay SP263 and 22C3 or 28-8. |
| Skov^30^ | 2017 | - High level of agreement between 22C3 and 28-8 (*R*^2^ 0.95). - OPA, NPA and PPA high for all cut-offs (1%, 5%, 10% and 50%). |
| Soo^47^ | 2018 | Considerable variation in TC staining. Lowest correlation between 28-8 and SP142 (*R*^2^ 0.25). Highest between 22C3 and E1L3N (*R*^2^ 0.71). |
| Tsao^48^ | 2018 | - Close approximation between best-fit curves of 22C3, 28-8 and SP263. - Lower sensitivity of SP142, higher sensitivity of 73-10. |
| Xu^50^ | 2017 | - Assay SP142 stained fewer TCs compared to 22C3. - Using the 22C3 scoring algorithm, к was 0.481 between assays. - Using the SP142 scoring algorithm, к was 0.324 between assays. |
| Abbreviations: IC=immune cell; ICC=intraclass correlation coefficient; κ=kappa; LDT=laboratory-developed test; NPA=negative percent agreement; OPA=overall percent agreement; PPA=positive percent agreement; TC=tumour cell.  For references see main manuscript reference list. | | |

| **Supplementary table 6.** Results from studies assessing inter-observer concordance of TC scoring. | | |
| --- | --- | --- |
| **First author** | **Year** | **Study results (inter-observer concordance)** |
| Brunnström^32^ | 2017 | Varying κ values for antibodies:   - 22C3 0.71-0.95; - 28-8 assay 0.80-0.93; - SP263 0.75-0.91; - SP142 0.81-0.96; - 28-8 (LDT) 0.80-0.95.   Number of differently classified cases significantly higher for 1% cut-off. |
| Chan^35^ | 2018 | - ICC highest for SP263 (0.967), then 22C3 (0.963), 28-8 (0.932) and last SP142 (0.916). - Higher agreement for 50% cut-off than for 1% cut-off. |
| Conde^37^ | 2018 | - High ICCs for all antibodies (assays SP263 and SP142 and E1L3N) in both cohorts. - Highest concordance for 50% cut-off. |
| Cooper^33^ | 2017 | Assay 22C3:   - 1% cut-off: OPA 84.2%, κ 0.68; - 50% cut-off: OPA 81.9%, κ 0.58. |
| Ilie^39^ | 2016 | High inter-observer agreement for all antibodies:   - SP263 OPA 98%, κ 0.976; - SP142 OPA 92%, κ 0.910; - 28-8 OPA 96%, κ 0.935. |
| Ilie^40^ | 2017 | - 1% cut-off: κ 1 for all antibodies (22C3 assay and LDTs); - 50% cut-off: κ 1 for both 22C3 LDTs, κ 0.99 for 22C3 assay. |
| Marchetti^43^ | 2017 | Higher κ for 22C3 and SP263 for 50% cut-off (κ 0.931 and 0.942) than for 1% cut-off (κ 0.754 and 0.798) |
| Munari^44^ | 2018 | Good concordance between pathologists:   - SP263 κ 0.73; - 22C3 assay κ 0.77; - 22C3 LDT κ 0.72. |
| Ratcliffe^23^ | 2017 | Assays 22C3, 28-8 and SP263:   - OPAs at cut-offs 10%, 25% and 50% were >85%; - OPAs lower at 1% cut-off (75.9%-77.0%). |
| Rehman^45^ | 2017 | SP142 LDT: ICC 94% |
| Rimm^24^ | 2017 | Assays 22C3 and 28-8 and E1L3N and SP142 LDT:   - ICCs between 0.83 and 0.88; - Agreement higher at 50% cut-off (κ 0.75) than at 1% cut-off (κ 0.54). |
| Roach^34^ | 2016 | Assay 22C3: OPA 92.7%, NPA 92.6%, PPA 92.8%. |
| Russell-Goldman^29^ | 2018 | - Very high agreement (ICC 0.96) for E1L3N. - Agreement higher at 50% cut-off than at 1% cut-off (98% vs. 79%). |
| Scheel^25^ | 2016 | - Training set: κ 0.50 for E1L3N, κ 0.49 for SP142. Higher concordance with use of dichotomous cut-off criteria (κ 0.61-0.80). - Validation set: moderate concordance levels for the assays 22C3, 28-8, SP263 and SP142 (κ 0.47-0.49). Higher concordance with use of dichotomous cut-off criteria (κ 0.59-0.80). |
| Tsao^48^ | 2018 | Assays 22C3, 28-8, SP263, SP142 and 73-10:   - ICC for glass slide reading 0.88-0.93. For digital reading 0.80-0.91; - High-level reliability at various cut-offs, especially 5%, 10%, 25% and 50% (κ > 0.7); - Slightly diminished reliability at 1% and 80% cut-off. |
| Vennapusa^49^ | 2018 | Assay SP142: OPAs of 92.7%, 93.8% and 93.5% for TC1/IC1, TC2/IC2, and TC3/IC3. |
| Abbreviations: IC=immune cell; ICC=intraclass correlation coefficient; κ=kappa; LDT=laboratory-developed test; NPA=negative percent agreement; OPA=overall percent agreement; PPA=positive percent agreement; TC=tumour cell.  For references see main manuscript reference list. | | |

| **Supplementary table 7.** Results from studies assessing inter-laboratory concordance of TC staining. | | |
| --- | --- | --- |
| **First author** | **Year** | **Study results (inter-laboratory concordance)** |
| Adam^31^ | 2018 | Concordance for assays 22C3, 28-8 and SP263: κ 0.79-0.94. |
| Marchetti^43^ | 2017 | - Assay 22C3: ICC 0.973 for 4 centres. - Assay SP263: ICC 0.986 for 4 centres. |
| Roach^34^ | 2016 | Assay 22C3: OPA 88.3%, NPA 90.3%, PPA 85.2%. |
| Scheel^46^ | 2018 | - 6-step scoring system: substantial concordance for assays 22C3, 28-8 and SP263 (κ 0.63-0.69), moderate concordance for LDTs (κ 0.43). - 3-step scoring system: nearly perfect concordance for assays 22C3, 28-8 and SP263 (κ 0.73-0.89), moderate concordance for LDTs (κ 0.5). |
| Vennapusa^49^ | 2018 | Assay SP142: inter-site agreement of 87.6%, 87.6% and 91.0% for TC1/IC1, TC2/IC2, and TC3/IC3. |
| Abbreviations: IC=immune cell; ICC=intraclass correlation coefficient; κ=kappa; LDT=laboratory-developed test; NPA=negative percent agreement; OPA=overall percent agreement; PPA=positive percent agreement; TC=tumour cell. For references see main manuscript reference list. | | |

**Supplementary data 1.** Supplementary results: concordance of IC staining and scoring.

*Inter-assay concordance*

Eleven studies assessed inter-assay concordance of immune cell (IC) staining (supplementary table 8). Many of these found poor agreement of IC staining between assays and/or LDTs^28, 31, 35-37^ or greater variability in staining pattern for ICs than for TCs^22, 50^. One study^39^ showed much higher concordance (κ 0.721) between assays SP263 and 28-8 than between assays SP142 and SP263 (κ 0.018) and between assays SP142 and 28-8 (κ 0.134). Similarly, Rimm et al.^24^ found that inter-assay concordance of IC staining greatly approved after exclusion of SP142 (increase of ICC from 0.27 to 0.80). The Blueprint phase 2 study^48^, moreover, reported comparable distributions of IC scores among 22C3, 28-8 and SP263, while antibody SP142 showed lesser staining of ICs compared with the other antibodies.

*Inter-observer concordance*

Seven studies assessed inter-observer concordance of IC scoring (supplementary table 8). All of these reported lower concordance values of IC scoring compared to TC scoring for all antibodies^24, 25, 29, 37, 39, 45, 48^. Only Conde et al.^37^ reported comparable concordance levels for both IC and TC scoring in a cohort of 40 patients, while also reporting lower concordance levels for IC scoring than for TC scoring in another (validation) cohort of 29 patients.

| **Supplementary table 8.** Results from studies assessing inter-assay and/or inter-observer concordance of IC staining/scoring. | | | |
| --- | --- | --- | --- |
| **First author** | **Year** | **Study results (inter-assay concordance of IC staining)** | **Study results (inter-observer concordance of IC scoring)** |
| Adam^31^ | 2018 | Poor OPA when comparing assays 28-8, 22C3 and SP263 and when comparing LDTs to these assays. | NA |
| Chan^35^ | 2018 | Low concordance for IC scoring between all assays (*R*^2^ 0.263-0.682). | NA |
| Cogswell^36^ | 2017 | Assay 28-8 detected PD-L1 positive ICs more frequently than E1L3N (15 vs. 7). | NA |
| Conde^37^ | 2018 | Correlation for ICs lower than for TCs, lowest correlation for comparisons involving SP142. | High ICCs for all antibodies (SP263, SP142, E1L3N) in discovery cohort (0.92-0.96), lower ICCs in validation cohort (0.76-0.81). |
| Hendry^28^ | 2017 | Poor overall and pairwise agreement between IC stainings (ICC 0.212). | NA |
| Hirsch^22^ | 2017 | Greater variability in IC staining than in TC staining between assays. Highest concordance between 22C3 and 28-8. | NA |
| Ilie^39^ | 2016 | - Poor agreement between SP142 and SP263 (к 0.018) or 28-8 (к 0.134). - Good correlation between SP263 and 28-8 (к 0.721). | Agreement for ICs lower than for TCs:   - SP142 OPA 81%, κ 0.786; - SP263 OPA 87%, κ 0.832; - 28-8 OPA 86%, κ 0.817. |
| Rehman^45^ | 2017 | NA | IC scoring much less concordant (ICC 27%) than TC scoring (ICC 94%). |
| Rimm^24^ | 2017 | ICC was 0.27 for comparison of 22C3, 28-8, E1L3N and SP142. ICC increased to 0.80 after SP142 exclusion. | ICCs for IC scoring much lower (0.17-0.23) than for TC scoring (0.83-0.33). |
| Russell-Goldman^29^ | 2018 | NA | - Moderate agreement (ICC 0.47). - Agreement higher at 10% cut-off than at 1% cut-off (77% vs. 75%). |
| Scheel^25^ | 2016 | NA | In both training set and validation set low concordance of IC scoring (mostly κ <0.2). |
| Scheel^46^ | 2018 | - Similar IC staining patterns for assay 22C3 and 28-8, more intense staining with SP263. - Distinct IC staining pattern with SP142. | NA |
| Tsao^48^ | 2018 | - Distribution of IC scores comparable among 22C3, 28-8 and SP263. - Greater and lesser staining of ICs by 73-10 and SP142. | Overall poor agreement (κ 0.11-0.28 for glass slide reading and κ 0.08-0.27 for digital reading). |
| Xu^50^ | 2017 | Variability in staining pattern greater for ICs than for TCs. Lower PD-L1 detection levels with SP142 compared to 22C3. | NA |
| Abbreviations: IC=immune cell; ICC=intraclass correlation coefficient; κ=kappa; LDT=laboratory-developed test; NA=not applicable; OPA=overall percent agreement; TC=tumour cell. For references see main manuscript reference list. | | | |
